# Image artifacts caused by incorrect bowtie filters in cone‐beam CT image‐guided radiotherapy

**DOI:** 10.1002/acm2.12888

**Published:** 2020-05-08

**Authors:** Yanan Cao, Tianjun Ma, Steven F. de Boer, Iris Z. Wang

**Affiliations:** ^1^ Department of Radiation Medicine Roswell Park Comprehensive Cancer Center Buffalo NY USA; ^2^ Medical Physics Graduate Program University at Buffalo Buffalo NY USA

**Keywords:** bowtie filters, CBCT, crescent artifacts, image artifacts, IGRT

## Abstract

Certain models of cone beam computed tomography (CBCT) image‐guided radiotherapy (IGRT) systems require manually placing the appropriate bowtie filter according to the relevant imaging protocol. Inadvertently using a wrong bowtie filter or no bowtie filter could cause unexpected image artifacts. In this work, CBCT image artifact patterns caused by different bowtie filter placement were evaluated. CBCT images of CT phantoms, that is, a Body Norm phantom, a Catphan® phantom and an anthropomorphic RANDO® phantom, were acquired at a Varian Trilogy® unit with an On‐Board Imager® (OBI) system. Three image acquisition protocols were evaluated. For Standard Head protocol, half‐fan bowtie and no bowtie filter were studied for comparison with the correct full‐fan bowtie acquisition. For Pelvis and Low‐Dose Thorax protocols, full‐fan bowtie and no bowtie were studied for comparison with the correct half‐fan bowtie acquisition. In addition, the possibility of reversed direction half‐fan bowtie was also discussed. All possible scenarios of bowtie filter misplacement caused distinct artifacts regardless of protocols. These artifact patterns are different from the characteristic crescent artifact when correct bowtie filter was placed. Based on the artifact patterns described in this study we recommend reviewing image artifacts at time of image acquisition. If unexpected artifacts appear in the CBCT images, one should verify the correct placement of the bowtie filter and retake the image if necessary. However, it should also be stressed that using a wrong bowtie filter or forgetting to place the bowtie filter can cause increased patient dose. It is always a good practice to verify the bowtie filter placement before acquiring CBCT images for image‐guided radiotherapy.

## 1 INTRODUCTION

Cone‐beam computed tomography (CBCT) is a common image modality of three‐dimensional target localization and patient positioning in image‐guided radiation therapy (IGRT). Bowtie filters are used in CBCT system to deliver a more uniform fluence across the field of view. Certain models of CBCT IGRT systems require manually placing the appropriate bowtie filter according to the relevant imaging protocol.^1^ If a wrong bowtie filter or no bowtie filter is used, it could cause degraded image quality, increased patient dose and unexpected image artifacts.^2^, ^3^ It has been previously reported that a crescent artifact occurs when only the bowtie filter is used; the artifact is reproducible independent of phantom, position, system, reconstruction, and standard corrections.^4^, ^5^ This study will focus on evaluating the different CBCT image artifact patterns caused by a wrong or no bowtie filter compared with artifacts from the correct bowtie filter. We intend to demonstrate a way to verify whether the bowtie filter has been correctly implemented or not by reviewing the reconstructed CBCT images.

## 2 METHODS

### 2.A Cone‐beam CT system and reconstruction algorithms

Investigations of image artifacts caused by different bowtie filter implemented in CBCT were performed on a Varian Trilogy® linear accelerator (Linac) equipped with an On‐Board Imager® (OBI) (Varian Medical Systems, Palo Alto, CA).^1^ Image data were acquired and reconstructed using OBI software 1.6, where FDK algorithm is used for reconstructing CBCT images from raw projection data. The reconstruction software uses two sets of projection data in CBCT image reconstruction, that is, one angle blank projection and hundreds of different angel object projections. For a 200‐degree gantry rotation, 360 projections were acquired and for 360‐degree gantry rotation 655 projections were acquired.

The OBI itself is a kilovoltage (kV) imaging system attached to the gantry at a 90‐degree offset from the megavoltage (MV) beam. The isocenter of OBI coincides with the Linac isocenter. The OBI consists of a kV x‐ray source and an amorphous silicon flat panel kV x‐ray detector. The kV source has a 0.4 and a 0.8 mm focal spots. The kV detector has an image size of 40 cm × 30 cm.^1^

**Fig. 1:**
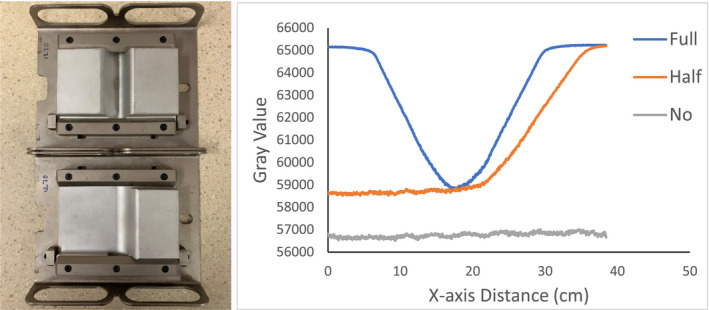
Left: Photo of bowtie filters (top: full‐fan, bottom: half‐fan). Right: The horizontal profiles through the center of the KV projection images.

OBI‐based CBCT can be acquired using either a full‐fan mode which corresponds to 200‐degree acquisitions or a half‐fan mode acquisition which corresponds to 360‐degree acquisitions. When the source to imager distance is at 150 cm and the detector is centered, the field of view of projection image is limited to 26 cm × 20 cm. Because the reconstructed field of view using full‐fan mode is limited to <24 cm in diameter, it will typically be used for small dimension body sections such as head and neck region. When the dimension of the imaged body section is larger, such as the pelvis, chest, and abdomen, the half‐fan acquisition mode should be used. In the half‐fan mode, the detector is shifted by 14.8 cm laterally with the blades in the kV source (kVS) cutting off approximately half of the beam aligning the beam axis at one edge of the detector.^6^ Either a full‐fan bowtie filter or a half‐fan bowtie filter for either of the fan modes can be mounted to the kVS to modify the beam profile for less patient dose and better image quality. Photos of these bowtie filters and the filter‐modified horizontal beam profiles through the center of the projection images are shown in Fig. 1.

### 2.B Image acquisition protocols

A variety of imaging protocols are available in the OBI system. In this study, three standard protocols were evaluated, that is, Standard Head (100 kVp, 145 mAs, 200‐degree gantry rotation), Pelvis (125 kVp, 680 mAs, 360‐degree gantry rotation), and Low‐Dose Thorax (110 kVp, 262 mAs, 360‐degree gantry rotation).

### 2.C Bowtie filter scenarios

**Fig. 2:**
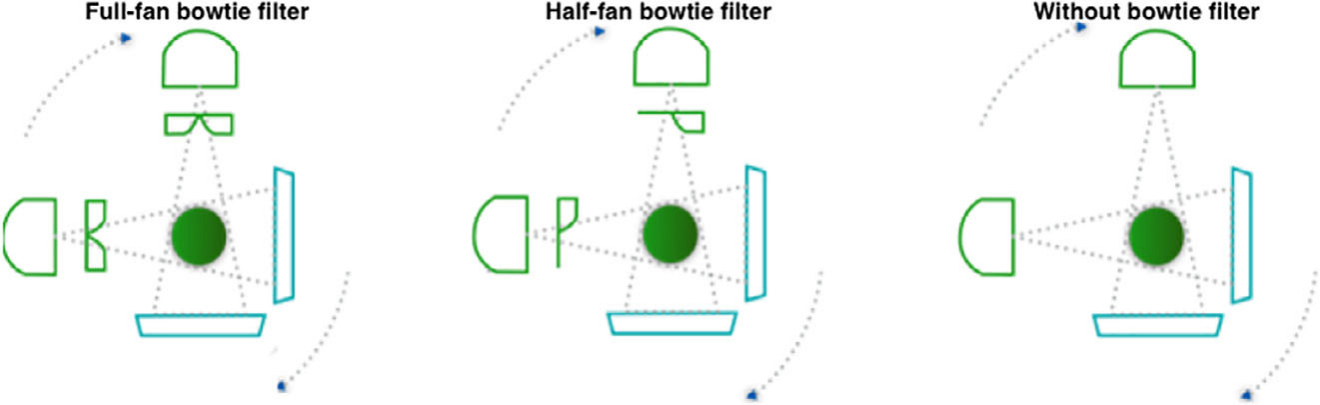
Standard Head scan protocols.

**Fig. 3:**
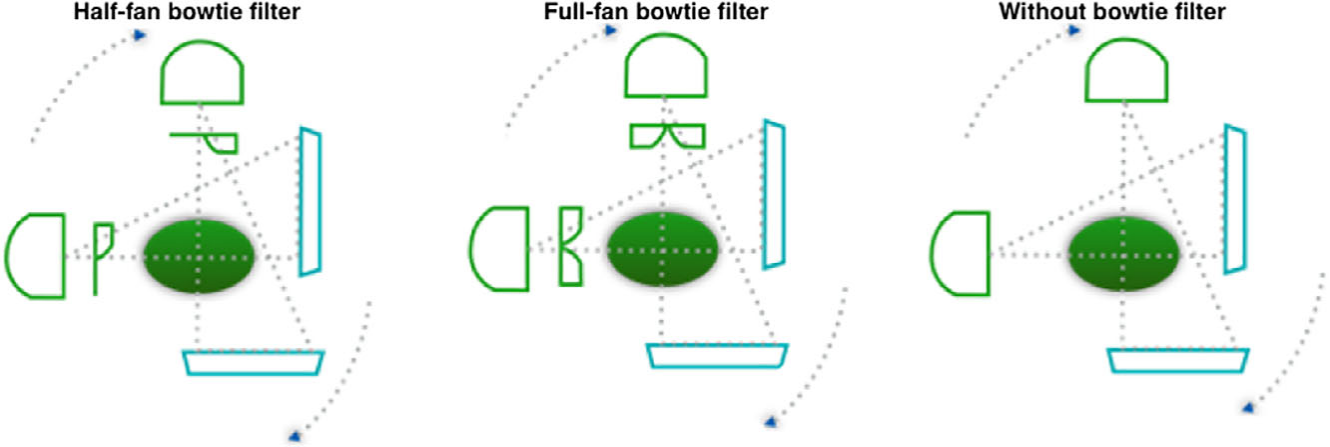
Pelvis and Low‐Dose Thorax scan protocols.

For Standard Head protocol, half‐fan bowtie and without any bowtie filter were studied for comparison with the correct full‐fan bowtie acquisition (Fig. 2). For Pelvis and Low‐Dose Thorax protocols, two scenarios were investigated, that is, full‐fan bowtie and without bowtie were studied for comparison with the correct half‐fan bowtie acquisition (Fig. 3).

### 2.D Phantoms

The uniform water section of a Catphan®phantom and the head of a RANDO phantom were used to evaluate image artifacts for Standard Head protocol. A Body Norm phantom and the torso of a RANDO phantom were used to evaluate image artifacts for Pelvis and Low‐Dose Thorax protocols.

## 3 RESULTS

**Fig. 4:**
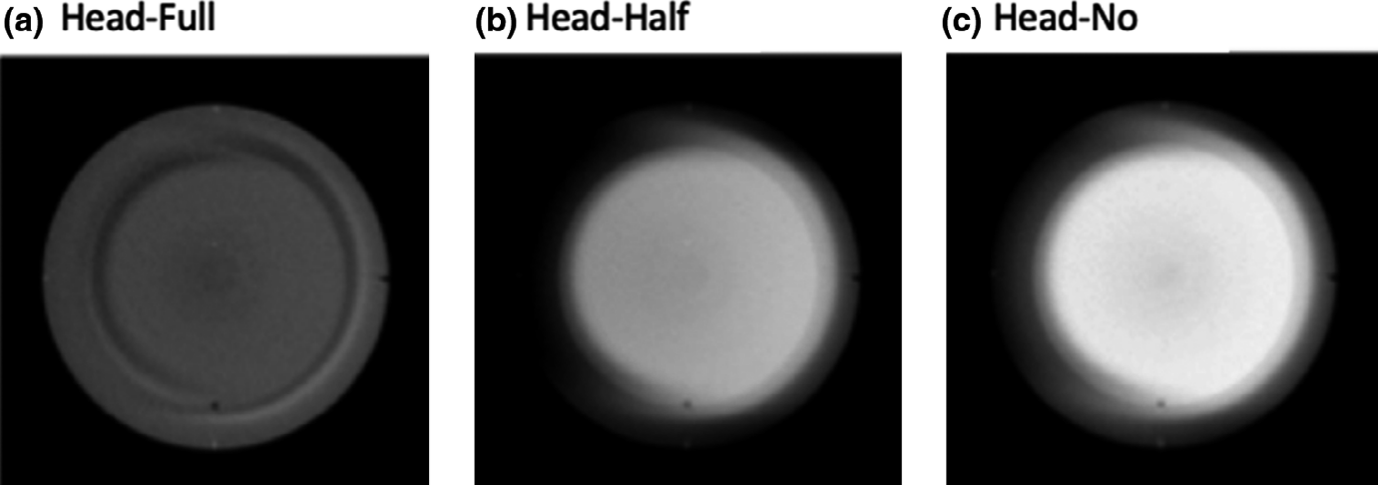
Trans‐axial cone beam computed tomography images of Standard Head protocol of Catphan phantom. All images were displayed with same window/level.

**Fig. 5:**
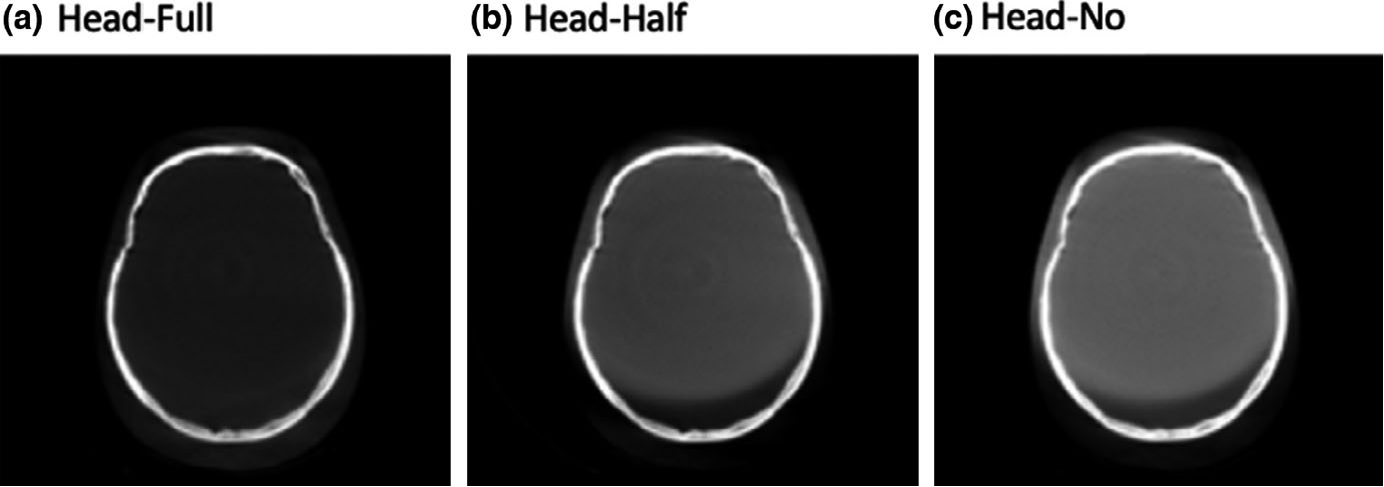
Trans‐axial cone beam computed tomography images of Standard Head protocol of a Head RANDO phantom. All images were displayed with same window/level.

Trans‐axial CBCT images of a uniform water‐equivalent portion of Catphan phantom and the head of a RANDO phantom, acquired utilizing Standard Head protocol, are shown in Figures 4 and 5. For the correct setup, that is, full‐fan bowtie filter placed, the image shows a crescent artifact [Figs. 4(a) and 5(a)], which is characterized by the dark and light bands in the image. The bands trace out a circle with a diameter of approximately 14 cm. When the half‐fan bowtie filter was placed instead, the images become brighter and brighter from left in the image to the right side [Figs. 4(b) and 5(b)]. When there is no bowtie filter placed, there is a capping artifact shown on the images [Figs. 4(c) and 5(c)], which is characterized by a brighter center with a diameter of approximately 14 cm.

**Fig. 6:**
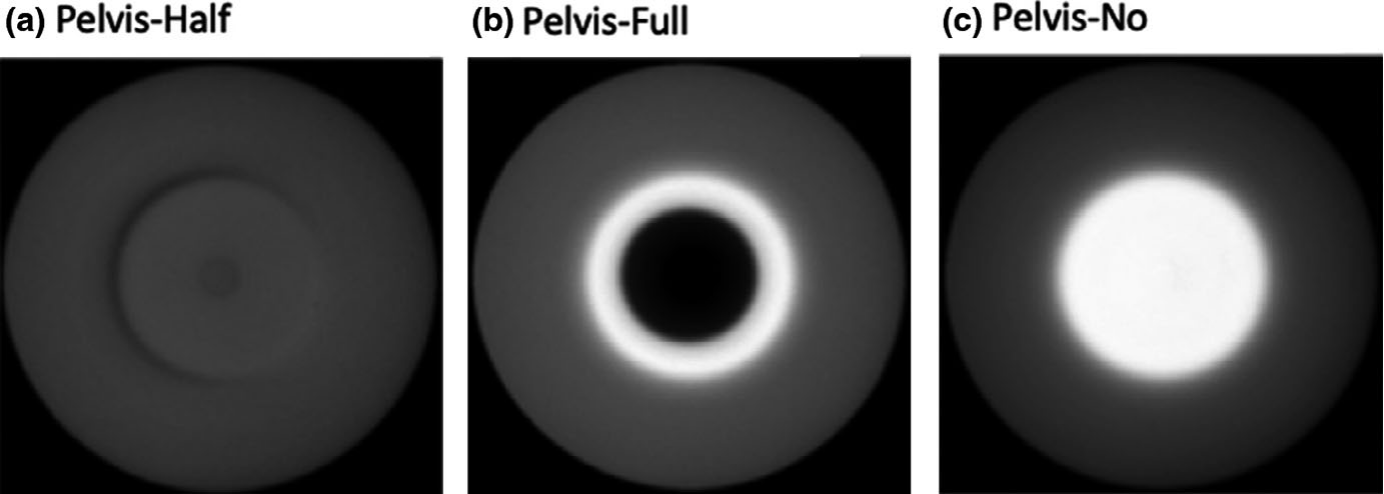
Trans‐axial cone beam computed tomography images of Pelvis protocol of a Body Norm phantom. All images were displayed with same window/level.

**Fig. 7:**
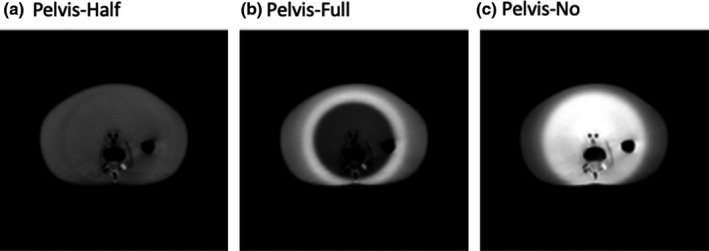
Trans‐axial cone beam computed tomography images of Pelvis protocol of a RANDO phantom’s torso. All images were displayed with same window/level.

**Fig. 8:**
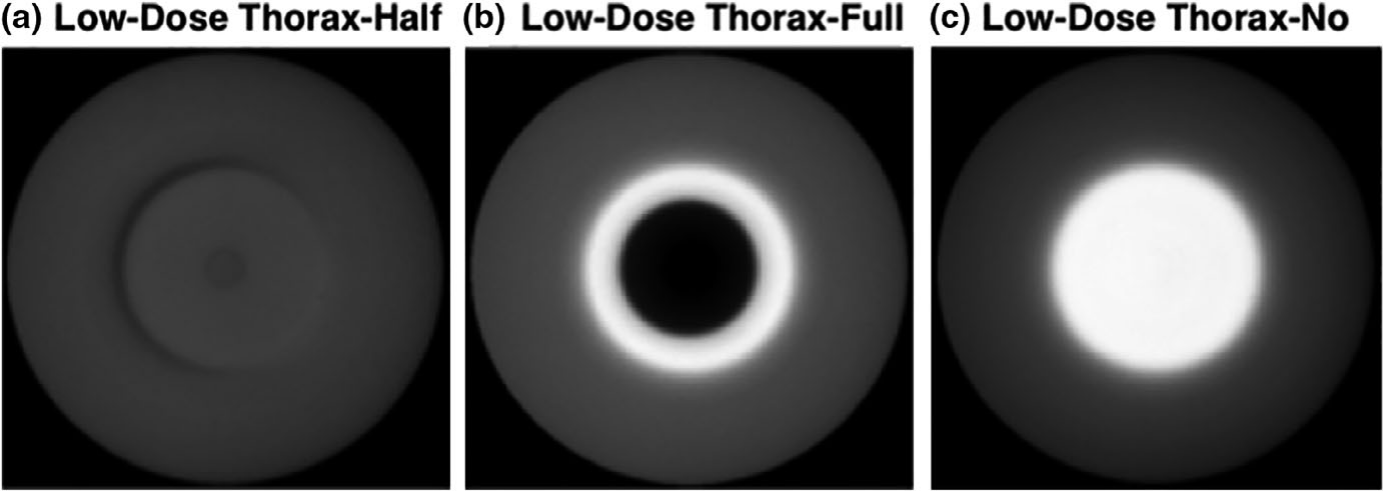
Trans‐axial cone beam computed tomography images of Low‐Dose Thorax of a body norm phantom. All images were displayed with same window/level.

**Fig. 9:**
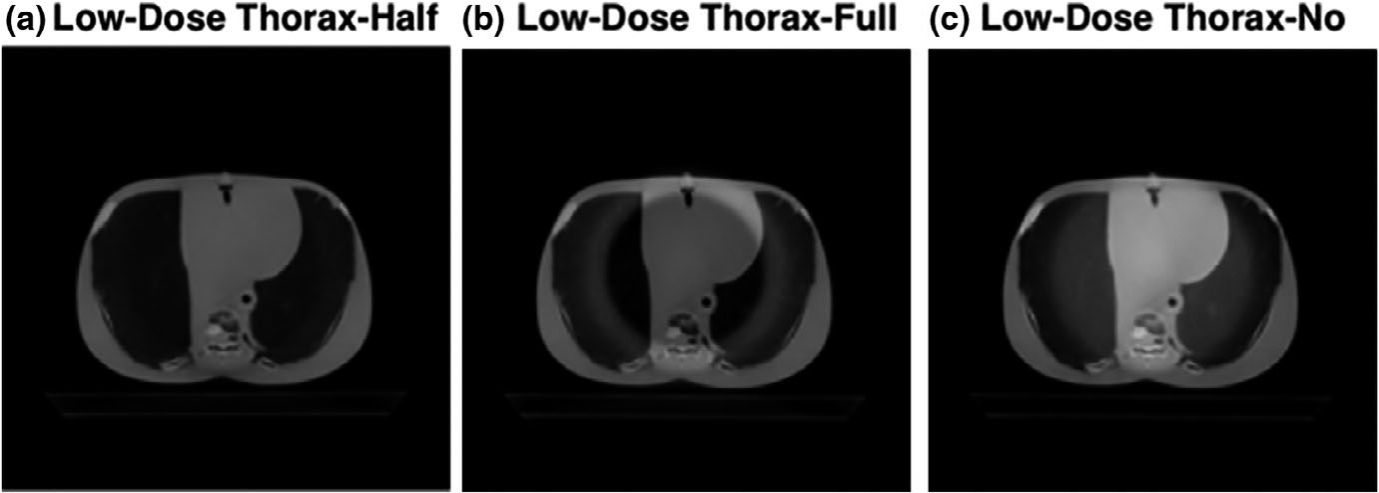
Trans‐axial cone beam computed tomography images of Low‐Dose Thorax of a RANDO phantom’s torso. All images were displayed with same window/level.

Trans‐axial CBCT images of a body norm phantom and the torso of a RANDO phantom, acquired utilizing either the Pelvis or the Low‐Dose Thorax protocols, are shown in Figs. 6, 7, 8, 9. For both of the pelvis and Low‐Dose Thorax protocols, the images show similar artifacts patterns. With the correct setup, that is, half‐fan bowtie filter placed, the images show a crescent artifact, which is characterized by the dark and light bands shown in Figs. 6(a)6, 7, 8, 9(a). The bands trace out a circle with a diameter of approximately 21 cm. When the full‐fan bowtie filter was placed instead, the images show an annular solar eclipse artifact that the brighter ring has at a diameter of approximately 18 cm for the inner ring as shown in Figs. 6(b)6, 7, 8, 9(b). When there was no bowtie filter placed, there is a capping artifact shown on the images, which is characterized by a brighter center with a diameter of approximately 18 cm as shown in Figs. 6(c)6, 7, 8, 9(c).

## 4 DISCUSSIONS

**Fig. 10:**
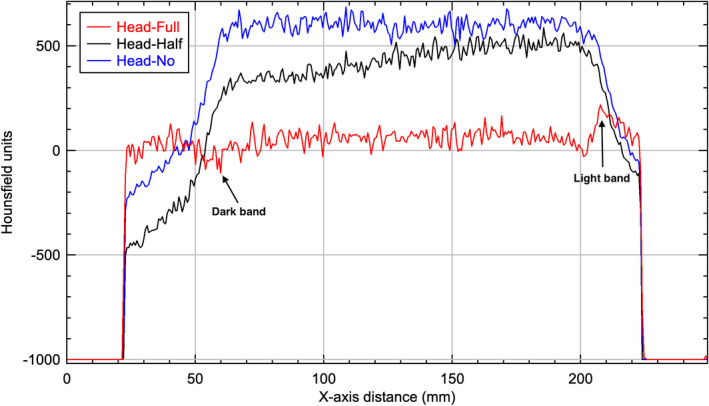
Horizontal profiles through the center of the reconstructed images of Standard Head protocol of Catphan phantom.

For all three protocols and with correct bowtie filter setup, the images show a characteristic feature of crescent artifacts. These crescent artifacts have been demonstrated and discussed previously in published literatures.^4^, ^5^ It was understood that either slight motions of the bowtie filter relative to the detector and the focal spot or slight motions of the bowtie filter and focal spot together relative to the detector could lead to an intensity mismatch between the all‐angle CBCT projections and a single‐angle blank projection. As a result, these motions caused the crescent artifacts observed in CBCT images when bowtie filter was used.^4^, ^5^ As the bowtie filter shifted in one direction, the incident fluence is higher on one side of the central ray and lower on the opposite side.^4^, ^5^ From the profiles through the centers of the reconstructed images of Standard Head protocol of Catphan phantom in Fig. 10 we can see this caused the attenuation in the corresponding voxels, as calculated by the reconstruction, to be higher or lower than it should be.^4^, ^5^ The end result was one light and one dark crescent shape (light and dark bands indicated in the full‐fan profile in Fig. 10) in the reconstructed image.

**Fig. 11:**
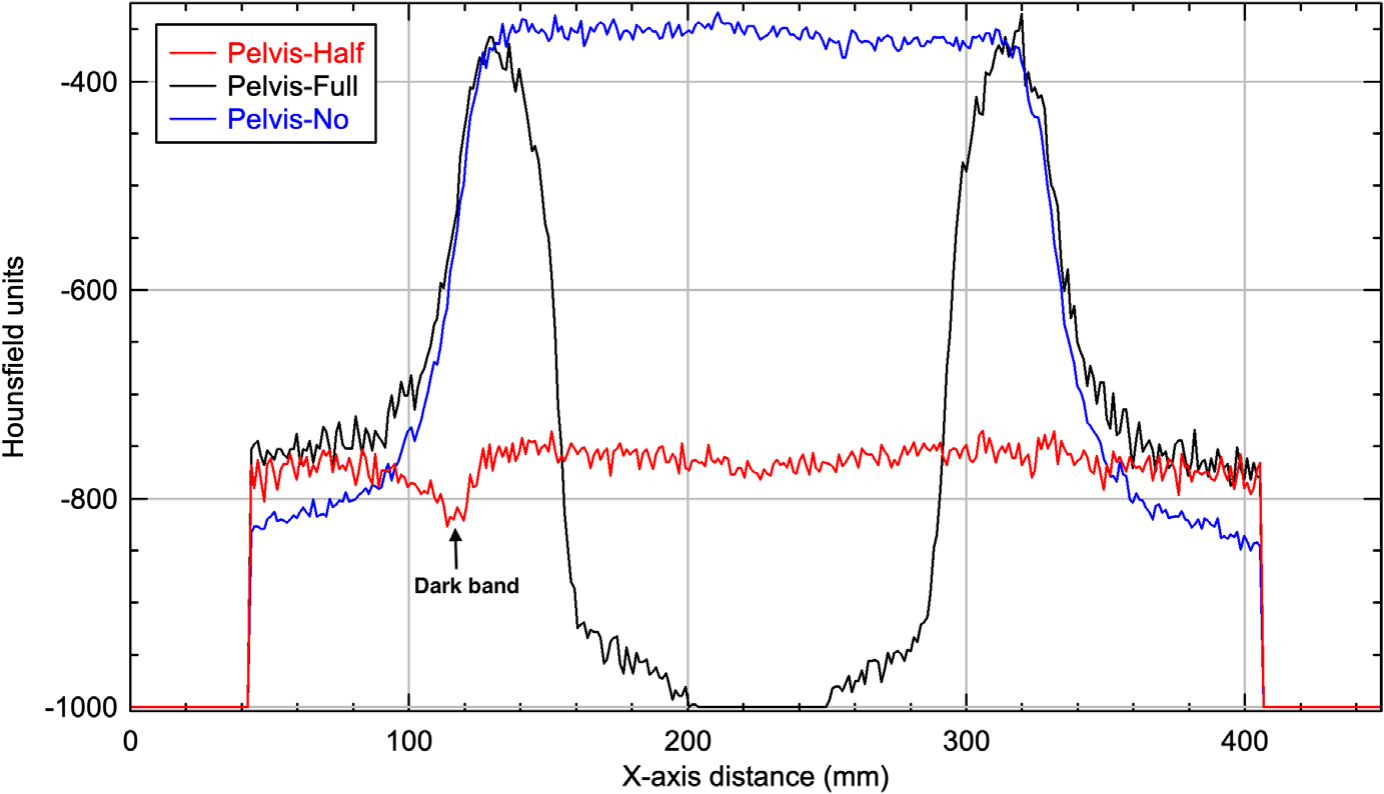
Horizontal profiles through the center of the reconstructed images of Pelvis protocol of Catphan phantom.

**Fig. 12:**
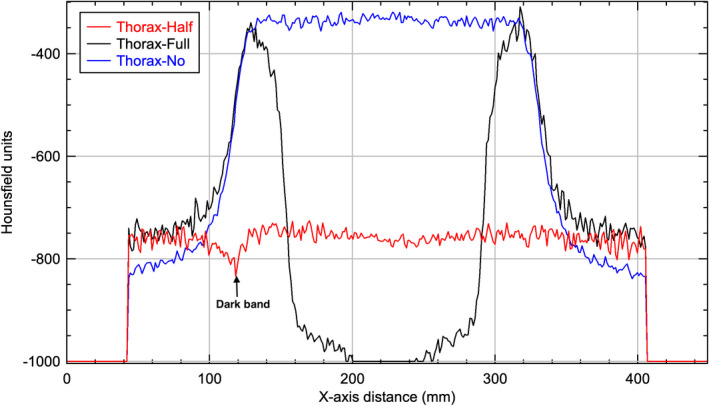
Horizontal profiles through the center of the reconstructed images of Low‐Dose Thorax protocol of Catphan phantom.

For the half‐fan bowtie filter, the crescent artifacts are less prominent as the relative shift in the filter position only affects one side of the fluence. If the filter is shifted with the thicker portion further from the central ray, the fluence will be higher along that ray creating an annulus of lower intensity (dark band indicated in Figs. 11 and 12 profiles). Conversely, if the filter is shifted with the thicker portion closer to the central ray, the fluence will be lower along that ray creating an annulus of higher intensity.

For all three protocols and with no bowtie filter setup, all images showed a capping artifact. The purpose of the bowtie filter is to attenuate more toward the periphery of the field in order to make the incident fluence at the detector more homogeneous.^7^ All the phantoms used in this study are circular in cross section which leads to an uncorrected incident fluence at the detector that is high at the edges and low in the center when no bowtie filter was in place. This caused the attenuation in the corresponding voxels, as calculated by the reconstruction, to be higher than it should be in the center and lower than it should be at the edge as seen in Figs. 10, 11, 12. The end result is a capping shape in the reconstructed image.

For the Standard Head protocol and with half‐fan bowtie filter setup, the reason the images become brighter from one side to another side is due to the difference of geometry design of the two bowtie filters. There is less attenuation material on one side of the half‐fan bowtie filter compared to the full‐fan bowtie filter. Therefore, the incident fluence is higher for the side with less attenuation material for half‐fan bowtie filter than that when the full‐fan bowtie filter was placed. From the profiles through the centers of the reconstructed images of Standard Head protocol of Catphan phantom in Fig. 10, we can see this caused the attenuation in the corresponding voxels, as calculated by the reconstruction, to be lower than it should be. The end results were as seen in Figs. 4(b) and 5(b).

For the Pelvis and Low‐Dose Thorax protocols and with full‐fan bowtie filter setup, the reason the images show an annular solar eclipse artifact is due to the difference of geometry design of the two bowtie filters as shown in Fig. 1. The full‐fan bowtie filter has a steeper gradient than the half‐fan bowtie filter and less attenuation in the center of the filter. When the full‐fan bowtie filter is placed, incident fluence across the detector is higher in the center and lower from the center to the edge than that when the half‐fan bowtie filter was placed. From the profiles through the centers of the reconstructed images of Pelvis and Low‐Dose Thorax  protocols of a Body Norm phantom in Figs. 11 and 12 we can see this caused the attenuation in the corresponding voxels, as calculated by the reconstruction, to be lower in the center region and higher in a ring region than it should be, respectively. The end results were as seen in Figs. 6(b)6, 7, 8, 9(b).

**Fig. 13:**
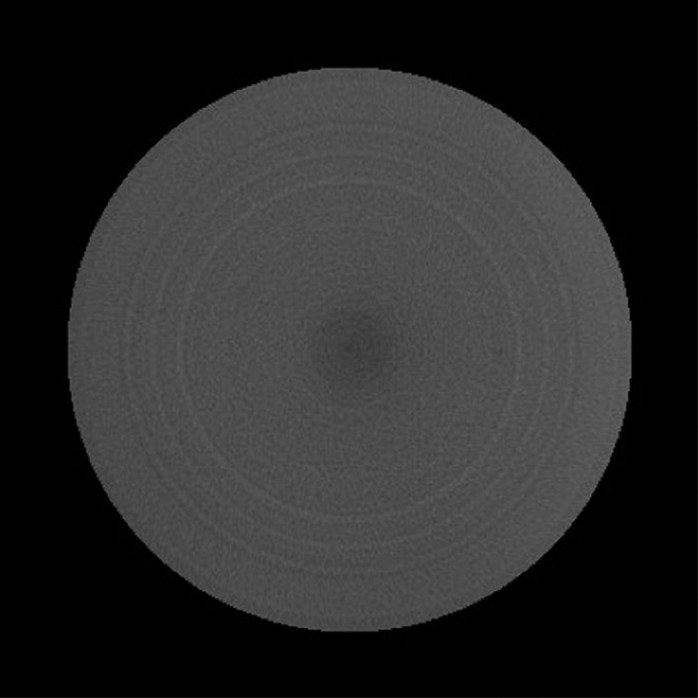
Trans‐axial cone beam computed tomography images of pelvis protocol with reversed half‐fan bowtie filter setup.

There is a special case one should pay attention to. For the Pelvis and Low‐Dose Thorax protocols, it is possible to have a reversed half (half‐R) bowtie filter setup. This scenario could occur directly from manufacturer and the user may not realize the error. In this case, the calibration procedure could have been carried out utilizing the reversed bowtie filter. In this setup, if the calibration has been carried out with the half‐R, the trans‐axial CBCT image (Fig. 13) would look more uniform than the correct half‐fan bowtie filter setup by human eyes. One should be also aware that because of the incorrect direction of the bowtie filter, there is additional patient dose associated with it. We measured the CTDI_w_ for Pelvis and Low‐Dose Thorax protocols with half‐fan bowtie filter and with half‐R bowtie filter for comparison. In these measurements, the scanning geometry, phantom size, beam quality, and mAs/projections were the same. The ratio of CTDI_w_ between the half‐R and the correct half‐fan bowtie filter are 1.56 and 1.59 for Pelvis protocol and Low‐Dose Thorax protocol, respectively. Therefore, the reversed half‐fan bowtie filter setup potentially could increase patient dose to more than 50%. Moreover, this setup actually plays a similar role like without bowtie filter placed which will decrease the image quality including uniformity, CT number accuracy, and contrast‐to‐noise ratio.^2^, ^3^

In summary, based on the artifact patterns described in this study we recommend reviewing image artifacts at time of image acquisition. If unexpected artifacts appear in the CBCT images, one should verify the correct placement of the bowtie filter and retake the image if necessary. However, it should also be stressed that using a wrong bowtie filter or forgetting to place the bowtie filter can cause increased patient dose. It is always a good practice to verify the bowtie filter placement before acquiring CBCT images for image‐guided radiotherapy.

## CONFILCT OF INTEREST

No conflict of interest.
